# Effectiveness of social network site-based interventions promoting condom use among youth: A systematic review based on the intervention mapping taxonomy

**DOI:** 10.1016/j.pmedr.2025.103318

**Published:** 2025-11-25

**Authors:** Iris de Visser, Marco Gaetani, Sophie Smith, Philippe Verduyn, Gill ten Hoor, Hanne M.L. Zimmermann

**Affiliations:** Maastricht University, Department of Work and Social Psychology, Maastricht, the Netherlands

**Keywords:** Condom, Young adult, Adolescent, Intervention, Social network sites, Behavioral change, Systematic review

## Abstract

**Objectives:**

This study aimed to systematically review existing interventions delivered via social network sites (SNS) to promote condom use among youth.

**Methods:**

Studies that evaluated SNS-based interventions targeting condom use and/or its psychosocial determinants among youth (12–24y) were searched. Intervention effectiveness was defined as percentage of comparisons (e.g., across different follow-up times or between different groups) showing significant improvements in condom use or determinants, out of total number of possible comparisons within each study. Using Intervention Mapping, determinants clusters targeted by each intervention, the behavioral change methods (BCMs) employed, and theoretical match between the targeted determinants and applied BCMs were inferred.

**Results:**

Fifteen studies were included. Median intervention effectiveness score was 33.3 % (IQR: 16.65,75 %) for condom use (*n* = 9) and 50 % (IQR: 0,100 %) for psychosocial determinants of condom use (*n* = 11). SNS-based interventions grounded in behavioral theory had a higher median effectiveness score when promoting condom use (50 % vs 33.3 %), but not when influencing its determinants (50 % vs 50 %). Two clusters of targeted determinants were identified: Attitude/Outcome Expectation/Risk Perception (*n* = 8) and Skills/Self-efficacy (*n* = 9), along with 25 distinct BCMs.

**Conclusion:**

While inconsistent reporting across studies limited identification of effective components, findings underline the importance of grounding interventions in behavioral theories.

## Highlights

• Targeting multiple determinants can enhance intervention effectiveness.
• Determinant and behavioral method match is important for intervention effectiveness.
• Targeting multiple behavioral determinants, enhances intervention effectiveness.
• Future studies should adopt standardized measures and improve transparency.
• There is need for further exploration of emerging social network sites.

## 1 Introduction

Sexually transmitted infections (STIs) pose a significant global public health concern ((WHO) WHO, 2024; *Lancet Child and Adolescent Health,* 2022). These infections can profoundly impact quality of life, resulting in adverse effects on individuals' physical, psychological, and social well-being (Shannon and Klausner, 2018). The urgency is also underscored by the substantial financial costs they impose on national healthcare systems (Chesson et al., 2021).

Several epidemiological data identified youth (i.e., 15–24 years) as the age group most exposed to the risk of contracting STIs, accounting for 53 % of new STIs cases in 2020 globally (*Lancet Child and Adolescent Health,* 2022; Shannon and Klausner, 2018). Since not all STIs are currently curable (e.g., HIV, HPV) and the prevalence of new infections transmitted through sexual contact is rising, preventive measures are crucial for reducing the spread of STIs ((WHO) WHO, 2024; *Lancet Child and Adolescent Health,* 2022; Organization WH, 2023). Condoms, when used correctly and consistently, represent one of the most effective methods of protection against STIs ((WHO) WHO, 2024). However, research shows the alarming trend in STIs diagnoses comes along with a significant decline in condom usage among sexually active youth (Koumans et al., 2020; Graaf et al., 2024).

Prior evidence grounded in behavioral theories showed condom use to be influenced by numerous psychosocial determinants, such as attitudes toward and intention to use condoms, perceived STI risk, peer norms, and skills (Krugu et al., 2016; Sheeran et al., 1999). Interventions aimed at promoting condom use can use different behavior change methods (BCMs) to target these psychosocial determinants of condom use and can be implemented via a diverse range of delivery channels (Eldredge et al., 2016). An emerging channel to deliver sexual health interventions is social network sites (SNS).

In recent years, SNS, such as Instagram, TikTok, and YouTube, have become an increasingly popular delivery platform for interventions, particularly among younger populations (Gabarron and Wynn, 2016; Engel, 2023). SNS are defined as “web-based services that allow individuals to construct a public or semi-public profile within a bounded system, articulate a list of other users with whom they share a connection, and view and traverse their list of connections and those made by others within the system” (Boyd and Ellison, 2007). SNS offer unique opportunities for health promotion, particularly among youth (Gabarron and Wynn, 2016; Gold et al., 2011; Plaisime et al., 2020). Given these features, SNS can serve as effective tools for promoting health, including condom use. Research has also shown that young individuals use SNS to seek health-related information (Burns et al., 2021), and their considerable popularity and interactive functionality make SNS powerful media tools for both health education and virtual dialogue within this population (Gold et al., 2011; Plaisime et al., 2020; Gabarron et al., 2014).

Although research supports the use of SNS as promising tools to deliver interventions aimed at promoting sexual health and condom use, the effectiveness of these SNS-based interventions remains poorly understood (Engel, 2023; Gold et al., 2011). Furthermore, there is limited insight into which behavioral determinants have been targeted, which behavior change methods have been employed, and whether these methods have been implemented under the appropriate conditions for effectiveness (i.e., parameters for effectiveness; (Kok et al., 2016)). Gaining deeper understanding of these key intervention elements is crucial for effectively designing, leveraging, and replicating SNS-based interventions (Eldredge et al., 2016; Gold et al., 2011).

Therefore, by using the Intervention Mapping (IM) taxonomy (Eldredge et al., 2016; Kok et al., 2016) as a shared language to describe the elements of interventions, this study aimed to systematically review the interventions delivered through SNS-based strategies to promote the use of condoms among youth, with a specific focus on evaluating their effectiveness, the behavioral determinants they targeted, the behavior change methods applied, and whether the parameters for effectiveness were met.

## 2 Methods

### 2.1 Search strategy

This systematic review was conducted in accordance with the Preferred Reporting Items for Systematic Reviews and Meta-Analyses guidelines (Page et al., 2021) (Table S6) and its protocol was pre-registered to assure transparency (PROSPERO, ref. CRD42024516694). A systematic search of published, peer-reviewed literature was performed via PubMed, Scopus, Web of Science, and PsycINFO.

The systematic electronic search was carried out in February 2024 and updated in October 2025. Studies were considered for inclusion if they were published from 1997 up to and including January 2024, as 1997 marks the launch of the first SNS available on the internet (Schaalma and Kok, 2009). The search strategy (Supplementary material, Table S1) included a combination of keywords directly related to predefined Population (Cavazos-Rehg et al., 2009), Intervention, Comparison, and Outcome (PICO), guided by the in- and exclusion criteria (Table S1).

Following the search, all identified papers were uploaded into Rayyan, an open-source tool for systematic review screening (Ouzzani et al., 2016). First, duplicates were removed, followed by title and abstracts screening against the eligibility criteria by two coders among IdV, MG and SS independently. Afterwards, full text screening was conducted. Studies were excluded in hierarchical manner. Non-primary literature (e.g., reviews, protocols) was first excluded, followed by studies not published in English. Next, studies were excluded in case the intervention was not delivered via SNS, did not focus on youth, or did not target condom use or its psychosocial determinants. In cases of discrepancies between coders, reasons were discussed, and a consensus decision was reached. If no consensus could be reached, the third coder was consulted to finalize the eligibility decision. Reference lists and citations of eligible studies were also screened to identify potential additional papers.

### 2.2 Eligibility criteria

Studies were eligible if they collected primary quantitative data through an experimental, quasi-experimental, or observational design. In addition, studies targeting adolescents or young adults (i.e., mean or median age of the sample included in the 12–24 range) were eligible. Gray literature and studies not making use of any SNS following the definition by Boyd and Ellison (Boyd and Ellison, 2007)) as a means of delivering the intervention were excluded. In addition, studies were eligible if the intervention targeted condom use-related behaviors (e.g. condom purchase, condom use) or psychosocial determinants of condom use-related behaviors (e.g. attitudes, social norms).

### 2.3 Data extraction

Data extraction was performed independently by one coder and cross-verified by at least another. The information extracted included study reference, participant demographics, intervention characteristics, the SNS used, and the outcomes of the intervention. Four coders (IdV, MG, GtH, HZ) analyzed the study materials for each eligible intervention to identify its underlying behavioral theory. Additionally, based on the interventions' information available, we used the IM framework (Eldredge et al., 2016; Kok et al., 2016) to infer the targeted determinants of condom use, the behavior change methods (BCMs) employed, and whether the parameters for effectiveness were met.

As specified in the protocol, the original aim was to systematically assess whether BCMs had been implemented under appropriate conditions for effectiveness (parameters for effectiveness), but the included studies did not report sufficient information to determine this. Consequently, a deviation from the original protocol was made, shifting focus to assessing theoretical alignment between the inferred psychosocial determinants targeted and BCMs used, in order to examine whether the interventions employed methods known to be effective in influencing the identified determinants (Eldredge et al., 2016; Kok et al., 2016). According to the IM taxonomy, some BCMs are basic individual-level methods (e.g., individualization, facilitation) that can be used to target multiple determinants, whereas other BCMs are more specific to certain clusters of determinants (e.g., planning coping responses for skills and self-efficacy). For the present analyses, knowledge was not considered a psychosocial determinant of condom use, given its association with other underlying fundamental constructs such as awareness, skills, self-efficacy, or social norms. Therefore, when a study reported targeting knowledge, the actual underlying psychosocial determinants and the BCMs addressing them were inferred. Definitions of each BCM are listed in Table S3. Authors independently coded each paper and subsequently discussed to reach an overall consensus.

### 2.4 Quality assessment

The quality of included studies was assessed independently by two reviewers using the Quality Assessment Tool for Quantitative studies by the Effective Public Health Practice Project (Thomas et al., 2008). Six key domains related to selection bias, study design, confounders, blinding, data collection methods, and withdrawals and drop-outs were considered. Each domain was given a rating of 1 (strong) to 3 (weak). Any discrepancies between the two reviewers were discussed to reach consensus, and a third reviewer was involved in case discrepancies persisted. Overall rating for each study was determined based on the mean rating of all domains. Studies were considered as “strong” in case no domain scored as weak, “moderate” in case one domain scored as weak, and “weak” in case of two or more domains scored as weak (Thomas et al., 2008).

### 2.5 Data analysis

For each study, an intervention effectiveness score was calculated. When a paper included multiple interventions, each was analyzed and counted as a separate unit. Effectiveness was defined as the percentage of comparisons showing a statistically significant difference in condom use or its psychosocial determinants, divided by the total number of possible comparisons within the study. Given that studies employed multiple outcome measures, at different follow-up times, and compared different groups, these were all treated as separate comparisons.

The effectiveness of each comparison was determined based on statistical evidence (i.e., *p*-values below the 0.05 threshold or confidence intervals not including 0 (for continuous outcomes) or 1 (for odds ratios)) indicating significant longitudinal changes within groups or cross-sectional differences between experimental conditions in condom use or its psychosocial determinants, as reported by the studies. For example, if a study included six subgroup comparisons and two showed significant improvements in the outcome, the resulting effectiveness score was 33.3 % (i.e., 2 effective comparisons out of 6 total possible comparisons). Comparisons showing a decrease in condom use or its determinants were considered as non-effective. If a study assessed the effectiveness of its intervention on both condom use and its psychosocial determinant(s), separate effectiveness scores were calculated for condom use and psychosocial determinants. These procedures followed the approach established in prior research (de Vries et al., 2023). Due to the small sample size (*n* = 15) and substantial heterogeneity among the included studies (in terms of study design, outcome measures, intervention components, and reporting quality), a meta-analysis could not be carried out. Instead, a narrative synthesis was conducted to summarize and interpret the findings.

## 3 Results

### 3.1 Study selection

**Fig. 1:**
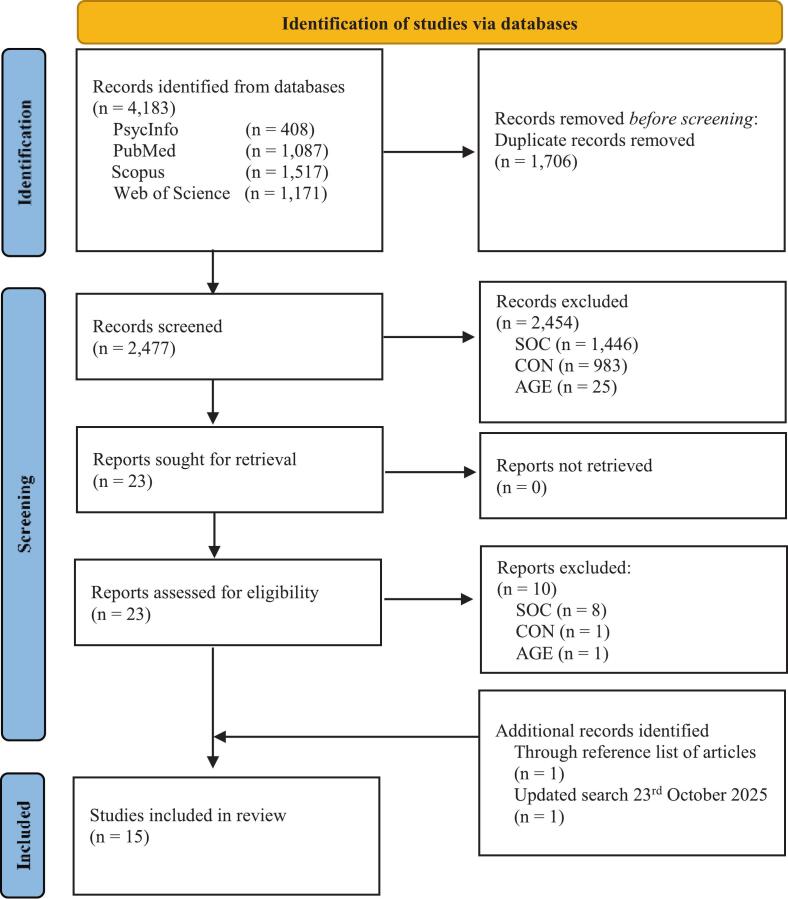
PRISMA flow diagram illustrating the search strategy for studies published from 1997 to 2025. *Note*. Only peer-reviewed published literature was considered.

In total, 15 studies were included. Fig. 1 provides an overview of the complete screening process and the reasons for exclusion of the articles. A total of 4183 articles were identified through database searches. After removing 1706 duplicate records, 2454 articles were excluded based on title and abstract screening. The most common reasons for exclusion were not using SNS for intervention delivery (*n* = 1466) and not reporting on condom-use outcomes (*n* = 983). Following full-text review of the remaining 23 articles, 13 studies were included. Additionally, one article was identified through reference list checking, and one article was included based on an updated search in October 2025 (*n* = 431 hits; *n* = 1 meeting our inclusion criteria).

Study characteristics are summarized in Table 1. All studies were published between 2012 and 2024. In total, ten were conducted in the Global North and five in the Global South. Twelve studies used an experimental design, while two were observational. The sample size for each study ranged from 33 to 1578 participants. The SNS used to deliver interventions were Facebook (*n* = 8), YouTube (*n* = 2), Instagram (n = 2), and Twitter (*n* = 1), other interactive platforms (*n* = 3), and private messages via several social media platforms (n = 1). The studies targeted a variety of populations, including specific age groups (e.g. college-aged (S9)), ethnicity (e.g. Hispanic, African-American, Asian, White (S10)), and sexual behaviors (e.g., MSM (S8,S11)). A comprehensive overview of the populations involved in the reviewed studies is provided in Table 1.

**Table 1:** Summary of main characteristics of studies delivering social network based interventions to promote condom use among youth (*n* = 15).

First author (year), country	Study design	Target population	Sample size (intervention / control)	Intervention (I) / Comparison or Control (C) condition	Type of SNS
Bull (2012), USA	Experimental	Mainly Hispanic, African-American, Asian, and white youth	*n* = 1578 (942 / 636)	**I:** Two months exposure to Just/Us Facebook page **C:** Two months exposure to a news Facebook page	Facebook
Clarke (2018), USA	Observational	Hispanic / Latin college students	*n* = 1406	**I:** Exposure to an HIV prevention campaign including: Free of charge HIV testing provided on campus and environmental campaign **C:** Baseline comparison	Facebook, Instagram, Twitter
Hightow-Weidman (2018), USA	Experimental	Young (age 18–30) Black MSM	*n* = 474 (238 / 236)	**I:** Exposure to interactive mobile phone and internet based intervention HealthMpowerment.org **C:** Exposure to an information-only website	Interactive theory-based website
Ko (2013), Taiwan	Quasi-experimental	MSM	*n* = 1037 (499 / 538)	**I:** Internet Popular Opinion Leaders (iPOL) disseminated HIV-related information on Facebook **C:** Informational control website	Facebook
Swendeman (2024), USA	Experimental	People between age 12–24	*n* = 985 (313 / 205 / 196 / 1181)	**I1**: Automated text messaging and monitoring (AMMI) **I2**: AMMI + peer support via private social media **I3**: AMMI + strengths-based telehealth coaching by near-peer paraprofessionals **I4**:AMMI + peer support and coaching	Private messages via social media
Neubaum (2014), Germany	Experimental	NA	*n* = 261 (133 / 128)	**I:** Exposure to a person-centered HIV blog **C:** Non-person centered HIV German governmental institutional website.	Blog
Boonkaewwan (2020), Thailand	Quasi-experimental	Adolescents living with HIV/AIDS between 10 and 19 years	*n* = 70 (35 / 35)	**I:** Usual care and nurse's support for adolescent HIV/AIDS sexual transmission prevention program via Facebook **C:** Usual care	Facebook
Prybutok (2013), USA	Experimental	18–24 year old students from undergraduate business course at University of North Texas	*n* = 33 (15 / 18)	**I:** Participants were shown an entertaining video about safe sex. **C:** Participants were shown a factual video about safe sex	YouTube
Jones (2012), USA	Observational	Adolescents and young adults aged 15–24 years	n = 70	All participants were shown a Facebook site	Facebook
Sun (2017), China	Experimental	Undergraduate students from Chinese universities aged younger than 25 years	*n* = 194 (96 / 100)	**I:** Six weeks of exposure to a private Facebook group developed with active youth input. **C:** Six weeks of exposure to an existing sexual health website.	Facebook
Hutchinson (2019), Kenya	Observational	Not specified	*n* = 700	**I:** One year exposure to the public-interest media platform Shujaaz **C:** No exposure to Shujaaz platform	Shujaaz platform (Facebook, Twitter, Instagram, YouTube, and Whatsapp)
Whiteley (2018), USA	Experimental	Youth from the age 15–24 with a history of vaginal or anal sex	n = 70 (31 / 29)	**I:** Exposure to publicly available web content **C:**Waitlist	YouTube
Fernandez (2019), USA	Experimental	Hispanic young adults between the age of 18–24 years	*n* = 60 (30 / 30)	**I:** Exposure to one of the campaign pages on either Facebook, Instagram or Twitter. **C:** No intervention.	Facebook, Twitter, Instagram
Young (2013), USA	Experimental	College-age individuals	*n* = 154 (79 / 75)	**I:** Exposure to sexually suggestive Facebook photos **C:** Exposure to nonsexual suggestive Facebook photos	Facebook
Bhandari (2024), Nepal	Quasi-experimental	Students between the age of 14–19 years from higher secondary schools	*n* = 160 (80 / 80)	**I:** Live quiz, E posters, info graphs, online discussion sessions delivered via Facebook messenger **C:** Basic education material	Facebook

*Note.* T = total; I = intervention group; C = Control group.

Participants' demographic characteristics are summarized in Table 2. Two studies included males or men only, while all the others included a mix of both females or women and males or men. Five studies did not specify the educational attainment of their sample, five studies had a mixture of educational attainments, four studies included university students only, and one higher secondary school students.

**Table 2:** Summary of sociodemographic characteristics (age, sex or gender, and educational attainment) and main outcomes related to condom use reported in studies included in this review of social network based interventions to promote condom use among youth (*n* = 15).

First author (year)	Age (yr) M ± sd	Sex or Gender: Female or women / Male / men (%) ^a^	Educational attainment, n (%)	Condom use related behavior	Psychosocial determinants of condom use related behavior
Bull (2012)	**I:** 19.8 ± 2.1 **C**: 20.2 ± 2.4	Not specified	NA	**Condom use last sex, %yes (95 % CI)** Baseline I: 0.65 (0.59, 0.71) vs C: 0.65 (0.58, 0.72) 2 month I: 0.68 (0.61, 0.74) vs C: 0.56 (0.48, 0.64) 6 month I: 0.60 (0.52, 0.98) vs C: 0.61 (0.52, 0.69) Time X condition interaction F = 3.30, *p* = 0.037 **Proportion of protected sex acts (95 % CI)** Baseline I: 0.63 (0.57, 0.68) vs C: 0.63 (0.58, 0.69) 2 month I: 0.62 (0.57, 0.68) vs C: 0.57 (0.51, 0.63) 6 month I: 0.55 (0.49, 0.62) vs C: 0.60 (0.54, 0.67) Time X condition interaction F = 3.63, *p* = 0.027	**Condom self-efficacy (95 %CI)** Baseline I: 3.38 (3.30, 3.46) vs C: 3.40 (3.31, 3.49) 2 month I: 3.42 (3.34, 3.50) vs C: 3.43 (3.33, 3.53) 6 month I: 3.41 (3.31, 3.50) vs C: 3.51 (3.42, 3.61) Time X condition interaction F = 0.95 *p* = 0.39 **Condom norms** Baseline I: 5.55 (5.36, 5.74) vs C: 5.49 (5.28, 5.70) 2 month I: 5.70 (5.49, 5.90) vs C: 5.75 (5.52, 5.97) 6 month I: 5.70 (5.47, 5.93) vs C: 5.83 (5.60, 6.06) Time X condition interaction F = 0.69, *p* = 0.50 **Condom intentions (%yes)** Baseline I: 0.87 (0.83, 0.90) vs C: 0.87 (0.83, 0.91) 2 month I: 0.87 (0.83, 0.91) vs C: 0.83 (0.77, 0.88) 6 month I: 0.85 (0.79, 0.90) vs C: 0.82 (0.75, 0.87) Time X condition interaction F = 1.27, *p* = 0.28
Clarke (2018), USA	20.4 ± 1.8	50.7 / 49.3	NA		**Perceived benefits of condom use** F (1, 632) = 4.96, *p* = 0.026 **Confidence in condom use** F (1, 631) = 3.97, *p* = 0.047
Hightow-Weidman (2018), USA	24.33 ± 3.22	0 / 100	< High school 43 (9.1) High school / GED, some technical / college 316 (66.7) College degree or more 115 (24.2)	**Condomless anal sex acts (past 3 months), M** ± **sd** Total sample: 6.11 ± 24.76 Intervention 5.74 ± 19.75 Control: 6.48 ± 28.97 **Condomless anal intercourse (CAI) incidence ratio (95 % CI)** 3 months: 0.63 (0.43, 0.93) 6 months: 1.14 (0.88, 1.94) 12 months: 1.09 (0.68, 1.50) **CAI incidence ratio (95 % CI) adjusted for loss to follow-up** 3 months: 0.74 (0.46, 0.99) 6 months: 1.42 (0.88, 1.95) 12 months: 1.11 (0.69, 1.53) **Intention-to-treat analysis** Intervention had 1.25 (0.34, 2.17) fewer CAI events at 3-month FU compared to control **Complier averaged causal effect** Treated observations 4.85 (2.15, 7.53) fewer CAI events at 3-month FU compared to the control group	
Ko (2013), Taiwan	T: 24.8 ± 6.2 I: 25.8 ± 6.3 C:23.8 ± 5.9	0 / 100	High school or less (%) I: 1 C: 1.8 College I: 65 C: 62 Post-college I: 20 C: 10	**No number of male sex partners with unprotected anal sex at baseline vs. follow-up, n (%)** I: 295 (59.23) vs. 279 (55.92) C: 302 (60.15) vs. 308 (57.25) X^2^ 2.3 with 3 df, *p* = 0.52 **Always use condom during anal sex with online sex partners at baseline vs. follow-up, n (%)** I: 68 (21.93) vs. 122 (37.54) C: 131 (36.49) vs. 137 (36.91) X^2^ 13.4 with 3 df, *p* = 0.004 **Always use condom during anal sex with male sex partners at baseline vs. follow-up, n (%)** I: 123 (34.65) vs. 81 (24.92) C: 100 (27.85) vs. 92 (24.79) X^2^ 3.0 with 3 df, *p* = 0.39	
Swendeman (2024), USA	T: 21.03 ± 2.15 I1: 21.05 ± 2.07 I2: 21.00 ± 2.27 I3: 20.88 ± 2.19 I4: 21.03 ± 2.15	7 / 93	Below high school, 137 (15 %) High school or equivalent 206 (23 %) Some higher education *n* = 419 (47 %) Completed higher education, 119 (13 %)	**Condom usage with all partners odds scale by intervention, OR estimate (95 % posterior interval for OR)** Visit 1.25 (1.10, 1.43) Visit x Coaching: 0.95 (0.86, 1.04) Visit x Peer support: 0.90 (0.82, 0.99) Visit x Peer support + Coaching: 0.95 (0.86, 1.05) Visit^2^: 0.98 (0.96, 1.00)	
Neubaum (2014), Germany	24.27 ± 3.71	61.7 / 38.3	96 % had at least university entrance-level qualification		**Attitudes toward condom and their use, M** ± **sd** I: 4.67 ± 0.34 vs C: 4.56 ± 0.35 t (259) = 2.53, *p* = 0.012 Cohen's d: 0.32 **Self-efficacy toward condom use, M** ± **sd** I: 4.31 ± 0.62 vs. C: 4.09 ± 0.68 t (259) = 2.77, *p* = 0.006 Cohen's d: 0.34 **Intention to use condom, M** ± **sd** I: 4.50 ± 0.75 vs. 4.46 ± 0.80 t (259) = 0.45, *p* = 0.65 Cohen's d: 0.05
Boonkaewwan (2020), Thailand	I: 15.74 ± 0.85 C: 16.00 ± 0.85	I: 40 / 60 C: 57.1 / 42.9	NA		**Behavioral intention for HIV/AIDS, median (IQR)** Baseline (I vs. C): 20 (9) vs. 20 (14) Day 15 (I vs. C): 10 (1) vs. 21 (16) Day 30 (I vs. C): 9 (3) vs. 25 (13) **Perceived effectiveness of HIV/AIDS preventative behaviors, median (IQR)** Baseline (I vs. C): 52 (18) vs. 55 (29) Day 15 (I vs. C): 34 (4) vs. 54 (33) Day 30 (I vs. C): 33 (6) vs. 49 (41)
Prybutok (2013), USA	Age 18–20 *n* = 3 (11.1 %) Age 21–24 *n* = 24 (88.9 %)	51.85 / 48.15	**Undergraduates** 26 (96.3) **Graduate students** 1 (3.7)		**Knowledge about safe sex, mean difference (variance)** Factual video: 2.07 (11.21) Entertaining video: 1.58 (5.00) *t* = 0.42, *p* = 0.67
Jones (2012), USA	**< 15 years** n = 1 (1 %) **15–17 years** *n* = 8 (11 %) **18–24 years** *n* = 61 (87 %)	70 / 30	NA	**Condom use, n (%)** Prior intervention 40 (57) After intervention 56 (80)	
Sun (2017), China	**17–18 years** *n* = 47 (24.2 %) **19–20 years** *n* = 85 (44.3 %) **21–22 years** *n* = 54 (27.8 %) **23–24 years** n = 8 (4.1 %)	67.5 / 32.5	All university students	**Condom use frequency (*n* = 40), M** ± **SD baseline vs. post intervention** I: 3.90 ± 1.34 vs. 4.10 ± 0.94 *p* = 0.51 C: 3.47 ± 1.58 vs. 3.31 ± 1.49 *p* = 1.49 Time X treatment analysis *p* = 0.29	**Condom use attitude, M** ± **sd baseline vs. post intervention** I: 3.15 ± 0.43 vs. 3.26 ± 0.44 *p* = 0.02 C: 3.24 ± 0.47 vs. 3.31 ± 0.39 *p* = 0.15 Time X treatment analysis *p =* 0.54 **Contraceptive use intention, M ± sd baseline vs. post intervention** I: 3.04 ± 0.52 vs. 3.15 ± 0.51 *p* = 0.08 C: 3.09 ± 0.68 vs. 3.05 ± 0.52 *p* = 0.52 Time X treatment analysis *p* = 0.10 **Perceived difficulty and ease of condom use (skills), M ± sd baseline vs. post intervention** I: 3.26 ± 0.58 vs. 3.49 ± 0.52 *p* ≤0.001 C: 3.32 ± 0.57 vs. 3.43 ± 0.57 *p* = 0.07 Time X treatment analysis *p* = 0.29
Hutchinson (2019), Kenya	Baseline 18.4 (se 2.6) Endline 19.9 (se 2.6)	43.7 / 56.3	None/Primary 13.7 % Primary 58.9 % Secondary 21.7 % Post-*sec* 5.7 %	**Ever use of condoms (%)** No exposure to digital media 50.5 <1 year exposure to digital media 58.8 *p* = 0.25 >1 year exposure to digital media 73.3 *p* = 0.003	**Recommend condom (%)** No exposure to digital media 42.7 < 1 exposure to digital media 45.1 *p* = 0.74 >1 exposure to digital media 68.9 *p* = 0.001
Whiteley (2018), USA	18.6	38 / 62	Not specified	**Unprotected vaginal / anal sex in past 3 months (% yes)** Pre-intervention I: 30 vs. C: 43 Post-intervention I: 17 vs. C: 48 AOR 7.77 *p* ≤0.05	**HIV related knowledge, M ± sd** Pre-intervention I: 56.7 ± 20.9 vs. C: 59.6 ± 12.6 Post-intervention I: 62.8 ± 19 vs. 61.6 ± 18.7 F (1.58) = 0.59 **HIV prevention self-efficacy, M ± sd** Pre-intervention I: 40.3 ± 6.5 vs. C: 42.4 ± 6.2 Post-intervention I: 44.8 ± 4.5 vs. C: 42.6 ± 6.1 F (1.58) = 5.71
Fernandez (2019), USA	T: 21 ± 1.95 I: 21 ± 1.98 C: 21 ± 1.95	T: 38 / 62 I: 46.7 / 53.3 C: 30 / 70	All university students	**Condom use with primary partner M ± sd,** Baseline I: 2.24 ± 1.44 vs. C: 1.63 ± 1.42 Follow-up I: 2.00 ± 1.64 vs. C: 2.17 ± 1.77 **Condom use with someone other than a primary partner, M ± sd** Baseline I: 1.50 ± 1.36 vs. C: 1.61 ± 1.50 Follow-up I: 1.30 ± 1.42 vs. 1.25 ± 1.58	**Confidence of using condoms at next sex act, M ± sd** Baseline I: 1.20 ± 0.48 vs. C: 1.26 ± 0.52 Follow-up I: 1.26 ± 0.52 vs. C: 1.26 ± 0.51 **Benefits of condom use during next sex act, M ± sd** Baseline I: 1.10 ± 0.31 vs. C: 1.10 ± 0.31 Follow-up I: 1.13 ± 0.34 vs. C: 1.16 ± 0.37
Young (2013), USA			All university students		**Personal likelihood of unprotected sex, M ± sd** I: 4.1 ± 2.0 vs. C: 3.4 ± 2.0 95 % CI (−1.4, −0,1) **Perceived peer prevalence of unprotected sex, M % ± sd** I: 36.7 ± 32.4 vs. C: 25.2 ± 22.7 95 % CI (−20.5, −2.5)
Bhandari (2024)	Age 14–19 years	Not specified	All higher secondary school students.		**STI related knowledge, M ± sd** Pre-intervention I: 2.33 **±** 1.41 vs. C: 2.14 ± 1.36 Post-intervention 1: 4.62 ± 1.56 vs. C: 2.10 ± 1.39 Post-test between groups *t* = 10.05, *p* = 0.001 **STI prevention attitude, M ± sd** Pre-intervention I: 21.87 **±** 3.25 vs. C: 22.31 ± 2.34 Post-intervention 1: 26.39 ± 4.33 vs. C: 20.12 ± 1.90 Post-test between groups *t* = 11.29, *p* = 0.001 **Subjective norms, M ± sd** Pre-intervention I: 13.93 **±** 2.29 vs. C: 14.50 ± 2.10 Post-intervention I: 17.59 **±** 2.23 vs. C: 14.67 ± 2.09 Post-test between groups *t* = 138, *p* = 0.001 **Perceived behavioral control, M ± sd** Pre-intervention I: 19.96 ± 4.13 vs. C: 20.94 ± 4.26 Post-intervention 1: 25.40 ± 4.41 vs. C: 21.12 ± 4.32 Post-test between groups t = 138, *p* = 0.001 **Intention, M ± sd** Pre-intervention I: 13.07 ± 3.26 vs. C: 13.79 ± 3.10 Post-intervention 1: 18.06 ± 3.02 vs. C: 12.35 ± 1.80 Post-test between groups t = 138, *p* = 0.001

*Note*. ^a^ Studies included in the paper measured either biological sex or gender identity, however this distinction was not always clearly reported. T = total; I = intervention group; C = Control group.

In total, four studies tested the impact of SNS-based interventions on condom use (S4,S7,S8,S11), six on the psychosocial determinants of condom use (S1,S5,S6,S9,S12), and five on both condom use and its psychosocial determinants (S2,S3,S10,S13,S14). Nine out of fifteen studies mentioned a behavioral theory or model underlying the intervention (Table 3).

**Table 3:** Summary of frequencies of health behavior theories or models, identified determinants, and identified behavioral change methods based on the Intervention Mapping Taxonomy across included studies in this review of social network based interventions to promote condom use among youth (*n* = 15).

Characteristic	n = 15 studies
**Health behavior theory or model, n**	
Information-Motivation-Behavioral Skills Model	2
Fishbein's integrative model of behavioral prediction	1
Integrated behavioral model	1
Pender's Health Promotion Model	1
Social Normative Theories	1
Theory of Planned Behavior	2
Trans Theoretical Model of Health Behavior	1
None mentioned	6
**Identified Determinants, n**	
Attitude / Outcome Expectation / Risk Perception	8
Self-efficacy / Skills	9
Unknown	5
**Behavioral Change Method, n**	
Discussion	8
Individualization	5
Use of Lay Workers & Peer Education	5
Facilitation	5
Cultural Similarity	4
Entertainment Education	4
Consciousness Raising	2
Fear Arousal	2
Mobilizing Social Networks	2
Modelling	2
Personalize Risk	2
Persuasive Communication	2
Self-reevaluation	2
Tailoring	2
Anticipated Regret	1
Belief Selection	1
Dramatic Relief	1
Framing	1
Mobilizing Social Support	1
Planning Coping Responses	1
Problem Posing Education	1
Providing Cues	1
Self-affirmation	1
Self-monitoring of behavior	1
Using Imagery	1
Unknown	1

Regarding the quality of the included studies, eight studies were considered as moderate and seven studies were rated as weak (Table S5).

### 3.2 Condom use(−related) behavior

Overall, nine studies assessed the effects of the interventions on condom use-related outcomes (Table 2). Three studies reported on condom use over specific time periods, such as the proportion of protected or unprotected sexual encounters during the last sex act (S10), over the past 60 days (S10), and over the past 3 months (S3,S8). Two studies used a frequency scale to quantify condom use, without a specified time frame (S11,S13). Two studies assessed condom use with a (primary) partner (S2,S7). Two studies measured the proportion of participants who (ever) used a condom (*n* = 2) (S4,S14).

The median effectiveness score of these interventions on condom use was 33.3 % (IQR 16.65 %,75 %, Table 4), indicating that the intervention with the median value of effectiveness significantly increased condom use in one out of three comparisons. Furthermore, interventions based on a behavioral theory (*n* = 5) had a median effectiveness score of 50 % (IQR 16.65 %,100 %), while those without any theoretical basis (*n* = 4) had a median effectiveness score of 33.3 % (IQR 16.65 %,33.3 %).

**Table 4:** Summary of Intervention Effects, Identified Determinants and Identified Behavioral Change Methods based on the Intervention Mapping Taxonomy.

First author (year)	SNS	Condom use		Psychosocial Determinants		Identified Determinants based on Intervention Mapping taxonomy^a^	Identified Behavioral Change Methods based on Intervention Mapping taxonomy
		Comparisons, n	Effectiveness	Comparisons, n	Effectiveness		
Bull (2012)	Facebook	6	33.3 %	9	0 %	Attitude / Outcome Expectations / Risk Perceptions Self-efficacy / Skills	Use of Lay Health Workers & Peer Education Mobilizing Social Support Discussion Entrainment Education
Clarke	Facebook, Instagram, Twitter			2	100 %	Attitude / Outcome Expectations / Risk Perceptions Self-efficacy / Skills	Cultural Similarity Facilitation
Hightow-Weidman (2018)	Interactive website	3	33.3 %			Knowledge is mentioned. Underlying determinants unknown.	Discussion Individualization Tailoring Personalize Risk Facilitation
Ko (2013)	Facebook	3	33.3 %			None	Use of Lay Health Workers & Peer education Mobilizing Social Networks Discussion Individualization
Swendeman (2024)	Private message via several SNS	3	33.3 %			None	Self-monitoring of behavior Self-reevaluation Self-affirmation Discussion Individualization Use of Lay Health Workers & Peer education
Neubaum (2014)	Blog			3	66.7 %	Attitude / Outcome Expectations / Risk Perceptions Self-efficacy / Skills	Fear arousal Anticipated Regret Personalize Risk Persuasive communication
Boonkaewwan (2020)	Facebook			4	50 %	Attitude / Outcome Expectations / Risk Perceptions Self-efficacy / Skills	Modelling Use of Lay Health Workers & Peer education Discussion Individualization Mobilizing Social Networks
Prybutok (2013)	YouTube			1	100 %	Attitude / Outcome Expectations / Risk Perceptions Self-efficacy / Skills	Persuasive communication Entertainment education Consciousness raising Using imagery Fear arousal
Jones (2012)	Facebook	1	100 %			Attitude / Outcome Expectations / Risk Perceptions Self-efficacy / Skills	Planning coping responses
Sun (2017)	Facebook	1	0 %	3	0 %	Attitude / Outcome Expectations / Risk Perceptions Self-efficacy / Skills	Discussion Individualization Use of Lay Health Workers & Peer education
Hutchinson (2019)	Interactive website	2	50 %	2	50 %	Self-efficacy / Skills	Discussion Modelling Cultural Similarity Framing Self-reevaluation Dramatic Relief Consciousness Raising Belief Selection
Whiteley (2018)	YouTube	1	100 %	2	50 %	Self-efficacy / Skills	Facilitation
Fernandez (2019)	Facebook, Twitter, Instagram	2	0 %	2	0 %	Attitude / Outcome Expectations / Risk Perceptions	Problem posing education Tailoring Entertainment education Cultural Similarity Facilitation Providing cues
Young (2013)	Facebook			2	100 %	None	None
Bhandari (2024)	Facebook			5	100 %	None	Facilitation Cultural similarity Entertainment education Discussion

*Note:*^a^The targeted determinants were based on the available information of the intervention in the article. In case it was unclear how the intervention targeted these determinants, we did not consider any targeted determinant.

In total, seven studies reported one or more statistically significant increases in condom use. Three of these studies used Facebook to deliver the intervention (S4,S10,S11). An interactive platform was used by two studies (S8,S14), and YouTube was used by one study (S3). One study delivered the intervention via private messages via several social media platforms, based on the participant's preference (S7). Two studies, one delivered via Facebook and the other via Facebook, Twitter and Instagram, found no significant increase in condom use (S2,S13).

### 3.3 Psychosocial determinants of condom use

Overall, eleven studies assessed the effects of the interventions on any psychosocial determinants of condom use (Table 2), namely, self-efficacy (*n* = 6), intention (n = 6), attitude (n = 6), safe sex knowledge (*n* = 3), and social norms (*n* = 4). The median effectiveness score of these interventions on the psychosocial determinants of condom use was 50 % (IQR 0,100 %), indicating that the intervention with the median effectiveness showed statistically significant improvements in the psychosocial determinants of condom use in half of its comparisons. Interventions based on a behavioral theory (*n* = 7) and those not based on any theory (n = 4) both had a median effectiveness score of 50 %, with IQRs of 50 %,100 % and 0 %,100 %, respectively.

Four of the six studies measuring self-efficacy reported a significant effect of the intervention. These interventions were delivered via YouTube (S3), a blog (S6), Facebook (S15), and a combination of Facebook, Twitter and Instagram (S9). Two studies delivered via Facebook found no significant changes in self-efficacy after the intervention (S10,S13).

Three of the six studies measuring condom use intention reported a significant difference in condom use intention between those exposed to the intervention and control participants. Interventions were delivered via a person-centered HIV blog (S6) and Facebook (S12,S15). Additionally, one study (S1) found that those exposed to sexually suggestive Facebook pictures reported higher personal likelihood of engaging in unprotected sex compared to participants who were exposed to nonsexual suggestive Facebook pictures. The remaining two interventions, both delivered via Facebook, found no significant difference in intention after the intervention (S10,S13).

Six studies reported on various outcomes related to condom use attitude, including attitude toward condoms and their use, perceived condom benefit, perceived effectiveness of HIV/AIDS preventative behaviors, and safer sexual behavior attitudes. Four studies detected a significant effect of the intervention on outcomes related to condom use attitude. These interventions were delivered via Facebook (S9,S12,S15), a person-centered HIV blog (S6) and Twitter and Instagram (S9). For two studies, both delivered via Facebook, no significant effect of the intervention on intention was detected (S2,S13).

Safe sex knowledge was investigated by three studies. Two studies showed significant increases in safe sex after intervention exposure. One study was delivered via YouTube (S5) and one via Facebook (S15). However, one study found no statistically significant difference in HIV-related knowledge between participants exposed to the publicly available web content, including YouTube, and those in the control group (S3).

Condom norms were investigated by four studies. This construct was measured by investigating condom norms (S10), proportion of participants who recommended condoms to friends and partners (S14), perceived peer prevalence of unprotected sex (S1), and subjective norms (S15). No significant effect of exposure to the Just/Us Facebook page compared to exposure to a news Facebook page (S10). One study found that those who had been exposed to the Shujaaz interactive platform for more than a year reported a higher percentage of recommending condom use to friends and partners, compared to those not exposed (S14). Furthermore, one study substantial improvements in subjective norms after exposure to the Facebook messenger intervention, compared to the control group (S15). Finally, one study found that viewing peer's Facebook pictures reduced perceived peer prevalence of unprotected sex, especially when exposed to less suggestive pictures (S1).

### 3.4 Targeted determinants, behavior change methods, and theoretical match based on the intervention mapping taxonomy

Two main clusters of targeted psychosocial determinants were identified across the included studies based on the IM taxonomy (Table 3). The cluster “Attitude/Outcome Expectation/Risk Perception” cluster appeared to be targeted in eight studies, while the “Skills/Self-efficacy” cluster was inferred in nine studies. In five studies, the targeted determinants could not be clearly identified based on the available intervention descriptions.

Using the IM taxonomy of behavior change methods (Eldredge et al., 2016), twenty-five different behavior change methods were identified (Table 3), with a median of four BCMs per intervention (IQR 2,5). The most frequently used (> 3) methods were Discussion (*n* = 8), Individualization (*n* = 5), Use of Lay Health Workers and Peer Education (n = 5), and Facilitation (n = 5). Tables S2 and S3 provide a comprehensive overview of the behavior change methods, their descriptions, parameters for effectiveness, frequency of use in the included studies, and the identified determinants and behavior change methods with supporting quotes from the articles.

For the determinant cluster Skills/Self-efficacy (*n* = 9), a median theoretical match (between determinants and BCMs) score of 50 % QR 25 %,75 %) was observed, meaning that, across the studies, half of the behavioral change methods matched the targeted determinants. Similarly, the determinant cluster Attitude/Outcome Expectation /Risk Perception (*n* = 8) showed a median theoretical match score of 55 % (IQR 25 %,87.5 %). No clear association emerged between the proportion of theoretical matches within interventions and their effectiveness scores (Table S4).

## 4 Discussion

This systematic review investigated the effectiveness of SNS interventions in promoting condom use and influencing its psychosocial determinants among young people. The median intervention effectiveness score indicated that, while some interventions achieved behavior change, the overall effectiveness of SNS-based interventions remained inconsistent. This key finding aligns with previous research reporting similarly mixed results (de Vries et al., 2023; von Sadovszky et al., 2014), and several possible explanations may account for this pattern.

Almost half of the included studies did not report using a theoretical framework to guide intervention development. Regarding condom use outcomes, interventions grounded in a behavioral theory showed higher median effectiveness compared to those not based on any theoretical framework. Concerning the psychosocial determinants of condom use, although median effectiveness scores for theory-based and non-theory-based interventions were the same, the IQR was narrower for interventions based on a theory, suggesting more consistent effectiveness of these interventions. Prior research emphasizes the role of theory in explaining behavior change and informing intervention design (Eldredge et al., 2016), with additional evidence suggesting greater effectiveness in interventions grounded in behavioral theories (Lopez et al., 2009; Kok, 2018; Cho YoonMin et al., 2018). Moreover, several scholars advocate for a multi-theoretical approach to adequately address the complexity of designing behavior change interventions promoting condom use (Kok, 2018; Schaalma and Kok, 2009).

Interventions targeting psychosocial determinants of condom use showed a higher overall median effectiveness score compared to those targeting condom use-related behaviors. This finding may be attributed to the complexity of condom use, which is shaped by multiple factors, including but not limited to the identified determinants. As such, achieving meaningful changes in actual condom use may be more challenging than modifying single psychosocial determinants. Among interventions for which targeted determinants could be inferred, two main clusters of determinants emerged: Skills/Self-efficacy and Attitude/Outcome Expectation/ Risk Perception. Although these determinants are known to be associated with condom use, focusing exclusively on one or few of them may be insufficient to achieve sustained behavior change. Future interventions may therefore benefit from addressing a broader range of determinants to enhance their effectiveness. Identification of relevant determinants to target can be improved by incorporating multiple behavioral theory frameworks during intervention design (Eldredge et al., 2016; Fernandez et al., 2019).

Most of the BCMs identified in the included studies were basic methods that can be leveraged to target multiple determinants simultaneously (e.g., discussion, individualization), rather than methods influencing specific determinants only (e.g., self-monitoring of behavior, anticipated regret). The use of basic BCMs may fit the passive engagement style of social media, since most techniques are designed for low-barrier engagement and are more generalizable. On the other hand, some of the determinant-specific BCMs, such as guided practice, require personalization and demand higher levels of structured interaction and (repeated) engagement, which can be more difficult to be achieved via SNS platforms. Even though SNS offer large reach and visibility, these platforms often lack the functionality to support the application of determinant-specific BCMs, potentially limiting their effectiveness. However, regardless of the methods employed, it is essential to assess their theoretical alignment with the targeted determinant, the relevance and modifiability of that determinant for the intended behavior change, and whether the parameters for effectiveness have been properly considered.

Results on the theoretical alignment between the targeted determinants and the applied BCMs indicated that approximately half of the BCMs were actually aligned with the determinants they intended to target. Although the selection of BCMs that are theoretically congruent with the targeted determinants is widely recognized as a critical aspect for intervention success (Eldredge et al., 2016; Abraham et al., 2014), this finding underscores the necessity for systematically considering such match during intervention development. Nevertheless, the theoretical match alone does not ensure intervention effectiveness, as the conditions under which BCMs are expected to be effective (i.e., parameters for effectiveness) must also be satisfied. However, since the included studies provided insufficient detail to determine whether these conditions were met, it was not possible to identify which components of the interventions contributed to their effectiveness and assess whether similar outcomes could be expected in other contexts (Eldredge et al., 2016).

### 4.1 Strengths, limitations and future directions

By applying the IM approach, the review systematically identified the targeted behavioral determinants and the BCMs implemented across the included interventions. This enabled a structured evaluation of how theoretically grounded and strategically designed the interventions were. Moreover, assessing the theoretical match between targeted determinants and applied BCMs clarified the internal logic underpinning these interventions.

Despite this, several limitations must be acknowledged. First, the lack of detailed reporting on intervention methodologies constrained the ability to fully evaluate theoretical coherence, map intervention logic, and identify the active components contributing to effectiveness. For example, some studies measured certain psychological determinants of condom use as measurement outcomes, but it was unclear based on the intervention's information, whether these determinants were also actively targeted by the intervention. Consequently, some targeted determinants may be underrepresented in our findings. This highlights the need for greater transparency in describing intervention content (Abraham et al., 2014; Borek et al., 2015; Davidson et al., 2003; Zogmaister et al., 2024; Altman et al., 2008).

Second, the small number of included studies combined with substantial heterogeneity in samples, outcome measures, follow-up periods, and intervention designs prevented a meta-analysis and limited the ability to identify which components contributed most to intervention effectiveness. This also precluded an evaluation of whether tailoring interventions to specific subgroups (e.g., sex, gender, age, ethnicity) improves outcomes, as suggested by prior research (de Vries et al., 2023; von Sadovszky et al., 2014).

While Facebook was the most commonly used SNS platform across the included studies, its popularity among youth has declined markedly in recent years. Future research should remain adaptive to the rapidly evolving SNS landscape to ensure interventions remain relevant and aligned with youth's online behaviors (Gabarron and Wynn, 2016; Gold et al., 2011; Yonker et al., 2015).

Lastly, the global decrease in condom use underlines the importance of developing effective interventions to promote condom use among youth. Given the widespread reach and interactive nature of SNS platforms, more research is needed to explore how these platforms can optimally be used to promote condom use. Additionally, more longitudinal studies are warranted to assess the long-term behavioral change.

## 5 Conclusion

This review found substantial inconsistency in the effectiveness of SNS-based interventions promoting condom use among youth, limiting the ability to draw firm conclusions. However, a key conclusion is that interventions grounded in behavioral theory tended to be more effective than those lacking a theoretical basis, underscoring the importance of designing interventions within established behavioral frameworks and ensuring stronger theoretical alignment between targeted determinants and applied BCMs.

## CRediT authorship contribution statement

**Iris de Visser:** Writing – review & editing, Writing – original draft, Visualization, Validation, Project administration, Methodology, Investigation, Formal analysis, Data curation. **Marco Gaetani:** Writing – review & editing, Writing – original draft, Visualization, Validation, Project administration, Methodology, Investigation, Formal analysis, Data curation, Conceptualization. **Sophie Smith:** Writing – original draft, Investigation. **Philippe Verduyn:** Writing – review & editing, Methodology. **Gill ten Hoor:** Writing – review & editing, Supervision, Project administration, Methodology, Conceptualization. **Hanne M.L. Zimmermann:** Writing – review & editing, Supervision, Project administration, Methodology, Conceptualization.

## Declaration of competing interest

The authors declare that there are no competing interests. This research was funded by the Fonds Wetenschappelijk Onderzoek Seksualiteit (FWOS; Fund for Scientific Research on Sexuality https://www.fwos.nl/). The funder had no role in the study design, data collection and analysis, decision to publish, or preparation of the manuscript.

## Data Availability

All available data can be found in the supplementary material.

## Appendix A Supplementary data

Supplementary material 1 Image 1
