# Supplementary material for: Effectiveness of social network site-based interventions promoting condom use among youth: A systematic review based on the intervention mapping taxonomy
Source: Prev Med Rep. 2025 Nov 25;60:103318. doi: 10.1016/j.pmedr.2025.103318 (PMC12712587; doi:10.1016/j.pmedr.2025.103318)
Supplement: Supplementary file 1 — Supplementary material 1 [file mmc1.docx]

​

**Supplementary material**

**Reference list of articles included in the systematic review**

1. Young SD, Jordan AH. The influence of social networking photos on social norms and sexual health behaviors. Cyberpsychology, behavior, and social networking. 2013;16(4):243-7.
2. Fernandez SB, Wagner EF, Howard M, Morris SL. Social media based strategies to reach Hispanic young adults with tailored sexual health information. Social Work and Social Sciences Review. 2019;21(1):73-93.
3. Whiteley LB, Brown LK, Curtis V, Ryoo HJ, Beausoleil N. Publicly available internet content as a HIV/STI prevention intervention for urban youth. The Journal of Primary Prevention. 2018;39:361-70.
4. Jones K, Baldwin KA, Lewis PR. The potential influence of a social media intervention on risky sexual behavior and Chlamydia incidence. Journal of community health nursing. 2012;29(2):106-20.
5. Prybutok G. YouTube: An effective web 2.0 informing channel for health education to prevent STDs. Informing Science. 2013;16:19.
6. Neubaum G, Krämer NC. Let’s blog about health! Exploring the persuasiveness of a personal HIV blog compared to an institutional HIV website. Health Communication. 2015;30(9):872-83.
7. Swendeman D, Rotheram-Borus MJ, Arnold EM, Fernández MI, Comulada WS, Lee S-J, et al. Optimal strategies to improve uptake of and adherence to HIV prevention among young people at risk for HIV acquisition in the USA (ATN 149): a randomised, controlled, factorial trial. The Lancet Digital Health. 2024;6(3):e187-e200.
8. Hightow-Weidman LB, LeGrand S, Muessig KE, Simmons RA, Soni K, Choi SK, et al. A randomized trial of an online risk reduction intervention for young black MSM. AIDS and Behavior. 2019;23:1166-77.
9. Clarke RD, Fernandez SB, Hospital M, Morris SL, Howard M, Wagner EF, Wales E. Getting their feet in the door: Communication cues to action for HIV testing and condom use behaviors among Hispanic/Latinx college students. The Journal of Primary Prevention. 2021;42(4):331-41.
10. Bull SS, Levine DK, Black SR, Schmiege SJ, Santelli J. Social media–delivered sexual health intervention: a cluster randomized controlled trial. American journal of preventive medicine. 2012;43(5):467-74.
11. Ko N-Y, Hsieh C-H, Wang M-C, Lee C, Chen C-L, Chung A-C, Hsu S-T. Effects of Internet popular opinion leaders (iPOL) among Internet-using men who have sex with men. Journal of medical Internet research. 2013;15(2):e40.
12. Boonkaewwan A, Punthmatharith B, Wiroonpanich W, Williams KA. Effects of a Nurse’s Support for the Adolescent-led HIV/AIDS Sexual Transmission Prevention Program using Social Media. Walailak Journal of Science and Technology (WJST). 2021;18(11):10010 (14 pages)- (14 pages).
13. Sun WH, Wong CKH, Wong WCW. A peer-led, social media-delivered, safer sex intervention for Chinese college students: randomized controlled trial. Journal of Medical Internet Research. 2017;19(8):e284.
14. Hutchinson P, Mirzoyants A, Leyton A. Empowering youth for social change through the Shujaaz multimedia platform in Kenya. International Journal of Adolescence and Youth. 2019;24(1):102-16.
15. Bhandari G, Dhital SR, Khatri D, Bhandari TR. Effectiveness of social media-based intervention in intention change of adolescents for promoting sexual health behavior in Western Terai of Nepal. Journal of Research in Health Sciences. 2024;24(2):e00613

**Table S1:** Full search strategy for the systematic review of social network based interventions to promote condom use among youth for included databases.

| **Key words** | **Search terms** |
| --- | --- |
| Social media | ((social media) OR (social network*) OR (digital*) OR (Facebook) OR (Instagram) OR (YouTube) OR (Snapchat) OR (TikTok)) |
|  | AND |
| Condom use | ((safe sex) OR (protected sex) OR (unprotected sex) OR (condom*) OR (prophylactic*) OR (condomless*)) |
|  | AND |
| Young age | ((adolescen*) OR (youth) OR (young*) OR (teen*) OR (students) OR (pupils)) |

Inclusion based on the PICO frame-work

- Population: young people (12-24 years old)
- Intervention: social network sites interventions aimed at promoting condom-use related behaviors and/or their psychosocial antecedents
- Control: compared to a comparison condition (e.g., control or other intervention group, pre-test measurements before receiving the intervention, waitlist control)
- All studies that collected data, at least pre- and post-intervention, are included, where at least our outcome (see next point) is measured.
- Outcome: condom-use related behaviors and their psychosocial antecedents.

**Table S2:** Extended summary of extracted data for behavioral change methods of studies included in the review, based on the Intervention Mapping Taxonomy

| **First author (year)** | **Theory mentioned used by the study** | **Determinants targeted by the intervention** | **Behavioral change methods** |
| --- | --- | --- | --- |
| Bull (2012) (42) | None | **Self-efficacy / Skills**  *Communication regarding sexual history, expectations for a healthy relationship, skills building for condom negation and condom use, and how to access STI testing*  **Attitude / Outcome Expectations / Risk perceptions**  *Expectation for healthy relationship; influences general sexual health risk* | **Use of lay workers & Peer education**  The use of youth facilitators |
|  |  |  | **Mobilizing social support**  The use of youth facilitators |
|  |  |  | **Discussion**  *“Threaded discussions relevant to week’s topic”* |
|  |  |  | **Entertainment education**  Quizzes and games |
| Clarke (2018) (41) | None | **Attitude / Outcome Expectations / Risk perceptions**  *Risk perception and stigma. Increasing awareness of risk (risk perception). Normalizing HIV testing as a health routine*  **Self-efficacy / Skills**  *Awareness of the resources and accessibility of HIV testing* | **Cultural similarity**  *“The language and graphics used were relatable for Hispanic / Latinx college population and in line with suggestions made by focus group participants”* |
|  |  |  | **Facilitation***  *HIV testing was offered at campus.*  *Should be noted that this is not delivered via SNS.* |
| Hightow-Weidman (2018) (40) | Integrated behavioral model (IBM) | **Knowledge**  The underlying determinants are however not clearly reported in the article. | **Discussion**  *“Forum for discussion of relevant topics such as HIV prevention and treatment, dealing with stigma, safer sex behaviors and relationships & space to upload and share personal videos, audio pictures or prose (Getting Real)”* |
|  |  |  | **Individualization**  *“The availability of an online doctor who would respond to questions within 72h / Decision support tool”* |
|  |  |  | **Tailoring**  *“The availability of an online doctor who would respond to questions within 72h / Decision support tool”* |
|  |  |  | **Personalize risk**  *“The availability of an online doctor who would respond to questions within 72h / Decision support tool”* |
|  |  |  | **Facilitation**  *Knowledge library / HIV testing locator* |
| Ko (2013) (43) | None | None | **Use of Lay Health Workers& Peer education**  *The use of iPOL / Have the capability to connect with other over the Internet for advice and support* |
|  |  |  | **Mobilizing Social Networks**  *The use of iPOL / Have the capability to connect with other over the Internet for advice and support* |
|  |  |  | **Discussion**  *Platform where they can discus and respond to questions* |
|  |  |  | **Individualization**  *Platform where they can discus and respond to questions* |
| Swendeman (2024) (39) | None | None | **Self-monitoring of behavior**  AMII*  Coaching step 4* |
|  |  |  | **Self-reevaluation**  AMII* |
|  |  |  | **Self-affirmation**  Coaching step 1* |
|  |  |  | **Discussion**  *Posting comments in a private online discussion, providing evidence-based responses links to information and referrals* |
|  |  |  | **Individualization**  *Posting comments in a private online discussion, providing evidence-based responses links to information and referrals* |
|  |  |  | **Use of Lay Health Workers & Peer Education**  The use of peer support |
| Neubaum (2014) (38) | Fishbein’s integrative model of behavioral prediction | **Attitude / Outcome Expectations / Risk perceptions**  *Risk perception: how one can be infected; definition of HIV and AIDS / transmission of HIV / HIV prevention*  **Self-efficacy / Skills**  *How to protect oneself or one’s partner against HIV* | **Fear arousal**  *“My immune system is attacked by the virus..” “I was infected with HIV when I had unsafe sex with my long-time girlfriend)* |
|  |  |  | **Anticipated regret**  *“My immune system is attacked by the virus..” “I was infected with HIV when I had unsafe sex with my long-time girlfriend)* |
|  |  |  | **Persuasive communication**  *“For me, it is important to motivate people to use condoms in risk situations”* |
| Boonkaewwan (2021) (44) | Theory of Planned Behavior | **Attitude / Outcome Expectations / Risk perceptions**  *Risk perception / attitude toward performing specific HIV / AIDS prevention behaviors*  *Safer-sex discussion / avoidance of risky behavior / condom accessibility*  **Self-efficacy / Skills**  *Risk perception / attitude toward performing specific HIV / AIDS prevention behaviors* | **Use of Lay Health Workers & Peer Education**  Peer educators |
|  |  |  | **Modelling**  *“Negotiation skills with short film VDOs that show situations where adolescents are using negotiation skills”* |
|  |  |  | **Discussion**  *“Adolescent leaders can chat, share, and conduct individual chats with adolescents”* |
|  |  |  | **Individualization**  *“Adolescent leaders can chat, share, and conduct individual chats with adolescents”* |
|  |  |  | **Mobilizing Social Networks**  Keep social contacts and continuously communicate with friends in the group. |
| Prybutok (2013) (37) |  | Knowledge is mentioned, however underlying determinants are unclear. What is based on the videos:  **Self-efficacy / Skills**  **Attitude / Outcome Expectations / Risk perceptions** | Factual video  **Persuasive communication**  In factual and entertaining video: Providing reinforcing and punishing messages with the aim to persuade the persone. |
|  |  |  | **Consciousness raising**  In both the factual and entertaining video |
|  |  |  | **Entertainment education**  The whole entertaining video |
|  |  |  | **Fear arousal**  Based on content of the video. |
|  |  |  | **Using Imagery**  Based on the entertainment video. |
| Jones (2012) (36) | Pender’s Health Promotion Model | **Self-efficacy / Skills**  *STD prevention and transference, including correct condom application and safe sex practices; and how to talk to one’s partner and parents once a diagnosis occurs*  **Attitude / Outcome Expectations / Risk perceptions**  *Myths and facts of chlamydia contagion / perceived benefits /Signs, symptoms, complications of Chlamydia infections.* | **Planning coping responses**  *“How to talk to one’s partner and parents once a diagnosis occurs”* |
| Sun (2017) (45) | Information- motivation-behavioral skills model | Knowledge is mentioned. However, the underlying determinants are unclear.  Based on the intervention the following determinants were identified:  **Self-efficacy / skills** | **Discussion / Individualization**  *“Where group members can post on the wall and interact through discussion threads and peer educators could respond to questions”* |
|  |  |  | **Use of Lay Health Worker & Peer Education**  The use of peer educators |
| Hutchinson (2019) (46) | Trans theoretical model of health behavior | **Self-efficacy / skills**  *Information to improve health, income, and overcome barriers* | **Discussion**  *“Where they can discuss pressing issues, especially sensitive or taboo topics, such as money, sex, relationships”* |
|  |  |  | **Modeling**  *“To tell stories of fictional characters and real people representative of youth from different parts of the country as they encounter and resolve challenging life and health issues”* |
|  |  |  | **Cultural Similarity**  *“To tell stories of fictional characters and real people representative of youth from different parts of the country as they encounter and resolve challenging life and health issues”* |
|  |  |  | **Framing**  *“Exposing participants to stories highlighting the relevance of proper planning on health-related issues in order to achieve their income and development goals; this is the stage of ‘norming’ contraception use ad an element of ‘smart planning’ & dispel myths and misconceptions”* |
|  |  |  | **Self-reevaluation**  *“Exposing participants to stories highlighting the relevance of proper planning on health-related issues in order to achieve their income and development goals; this is the stage of ‘norming’ contraception use ad an element of ‘smart planning’ & dispel myths and misconceptions”* |
|  |  |  | **Dramatic Relief**  *“Exposing participants to stories highlighting the relevance of proper planning on health-related issues in order to achieve their income and development goals; this is the stage of ‘norming’ contraception use ad an element of ‘smart planning’ & dispel myths and misconceptions”* |
|  |  |  | **Consciousness Raising**  *“Exposing participants to stories highlighting the relevance of proper planning on health-related issues in order to achieve their income and development goals; this is the stage of ‘norming’ contraception use ad an element of ‘smart planning’ & dispel myths and misconceptions”* |
|  |  |  | **Belief Selection**  *“Exposing participants to stories highlighting the relevance of proper planning on health-related issues in order to achieve their income and development goals; this is the stage of ‘norming’ contraception use ad an element of ‘smart planning’ & dispel myths and misconceptions”* |
| Whiteley (2018) (35) | Information Motivation and Behavioral skills model | **Self-efficacy / Skills**  *Motivation to stay safe and take necessary actions; behavioral skills for safe sex* | **Facilitation**  *“Links are send to the participants”* |
| Fernandez (2019) (34) |  | **Attitude / Outcome Expectations / Risk perceptions**  Reasons of unprotected sex / myths and facts | **Problem Posing Education**  *“The campaign also posed questions, encouraging users to respond and promoting community conversations”* |
|  |  |  | **Tailoring**  *“Posted time relevant and location relevant content”* |
|  |  |  | **Entertainment** **education**  *“Humor / multimedia posts such as videos with audio / memes / non-threatening”* |
|  |  |  | **Cultural** **similarity**  *“Developmentally revelant …. Targeting Hispanic Youth adults”* |
|  |  |  | **Facilitation**  *“Quick links / links to other websites”* |
|  |  |  | **Providing** **cues**  *“Weekly turnup tips”* |
| Young (2013) (33) | Social normative theories | None | None |
| Bhandari (2024) (47) | Theory of planned behavior | None | **Facilitation**  *“The intervention group received a social media-based health education intervention package, including educational materials, infographics, quizzes, e-posters, and interactive activities”*  **Cultural similarity**  *“These materials were presented in a culturally appropriate and language-friendly manner to ensure easy understanding and engagement”*  **Entertainment education**  *“quizzes / fun facts”*  **Discussion**  “*and interactive discussion activities”* |

Table S3: Summary of identified Intervention Mapping methods and parameters for effectiveness in based on the Intervention Mapping Taxonomy

| **Method** | **Method Description** | **Parameters** | **How many times used** |
| --- | --- | --- | --- |
| Anticipated Regret | Stimulating people to focus on their feelings after unintended risky behavior, before losses actually materialize. | Simulation of imagery; assumes a positive intention to avoid the risky behavior. | 2 |
| Belief Selection | Using messages designed to strengthen positive beliefs, weaken negative beliefs, and introduce new beliefs. | Requires investigation of the current attitudinal, normative and efficacy beliefs of the individual before choosing the beliefs on which to intervene. | 1 |
| Consciousness Raising | Providing information, feedback, or confrontation about the causes, consequences, and alternatives for a problem or problem behavior. | Can use feedback and confrontation; however, raising awareness must be quickly followed by increase in problem-solving ability and (collective) self-efficacy. | 2 |
| Cultural Similarity | Using characteristics of the target group in source message, and channel. | Using surface characteristics of the target group enhances receptivity. Using social-cultural characteristics leads to a more positive reception of the message. | 3 |
| Discussion | Encouraging consideration of a topic in open informal debate. | Listening to the learner to ensure that the correct schemas are activated. | 8 |
| Dramatic Relief | Encouraging emotional experiences, followed by reduced affect or anticipated relief if appropriate action is taken. | Preferably should be done in counseling context so that emotions can be aroused and subsequently relieved. | 1 |
| Entertainment Education | Providing a form of entertainment designed to educate (about health behavior) as well as to entertain. | Consideration of source and channel; balance of media professional’s and health promotor’s needs. | 4 |
| Facilitation | Creating an environment that makes the action easier or reduces barrier to action. | Requires real changes in the environment instead of in the perceptions of the environment. Requires the identification of barriers and facilitators and the power for making the appropriate changes. Facilitating conditions on one environmental level are usually dealt with by intervening on a higher environmental level. | 5 |
| Fear Arousal | Arousing negative emotional reactions in order to promote self-protective motivation and action. | Requires high self-efficacy expectations rather than high outcome expectations alone; is rarely effective. | 2 |
| Framing | Using gain-framed messages emphasizing the advantages of performing the healthy behavior; or loss-framed messages, emphasizing the disadvantages of not performing the healthy behavior. | Requires high self-efficacy expectations. Gain frames are more readily accepted and prevent defensive reactions. | 1 |
| Individualization | Providing opportunities for learners to have personal questions answered or instructions paced according to their individual progress. | Personal communication that responds to a learner’s needs. | 5 |
| Mobilizing Social Networks | Encouraging social networks to provide informational, emotional, appraisal, and instrumental support. | Availability of social network and potential support givers. Will often include information about others’ approval, facilitation, and persuasive communication. | 2 |
| Mobilizing Social Support | Prompting communication about behavior change in order to provide instrumental and emotional social support. | Combines caring, trust, openness, and acceptance with support of the behavioral change; positive support is available in the environment. | 1 |
| Modelling | Providing an appropriate model; being reinforced for the desired action. | Attention, remembrance, self-efficacy, skills, reinforcement of model; identification with model; coping model instead of mastery model. | 2 |
| Personalize Risk | Providing information about personal costs or risks of action or inaction with respect to target behavior. | Present messages ad individual and undeniable, and compare them with absolute and normative standards. | 3 |
| Persuasive Communication | Guiding individuals and environmental agents toward the adoption of an idea, attitude, or action by using arguments or other means. | Messages need to be relevant and not too discrepant from the beliefs of the individual; can be stimulated by surprise and repetition; will include arguments. | 2 |
| Planning Coping Responses | Getting the person to identify potential barriers and ways to overcome these. | Identification of high-risk situations and practice of coping response. | 1 |
| Problem Posing Education | Participatory analysis using critical reflection, self-disclosure, and dialogue regarding the social forces underlying a problem and a commitment to change self and community. | A safe environment for participation and disclosure; a critical stance. | 1 |
| Providing Cues | Assuring that the same cues are present at the time of learning and the time of retrieval. | Cues work best when people are allowed to select and provide their own cues. | 1 |
| Self-reevaluation | Encouraging combining both cognitive and affective assessments of one’s self-image with and without an unhealthy behavior | Stimulation of both cognitive and affective appraisal of self-image. Can use feedback and confrontation; however, raising awareness must be quickly followed by increase in problem-solving ability and self-efficacy. | 2 |
| Self-affirmation | Increasing people’s self-image by having them elaborate on their relevant values or desirable characteristics. | Must be tailored to individual self-image. | 1 |
| Self-monitoring of behavior | Prompting the person to keep a record of specified behavior(s). | The monitoring must be of the specific behavior (that is, not of a physiological state or health outcome). The data must be interpreted and used. The reward must be reinforcing to the individual. | 1 |
| Tailoring | Matching the intervention or components to previously measured characteristics of the participants. | Tailoring variables or factors related to behavior change (such as stage) or to relevance (such as culture or socioeconomic status) | 2 |
| Use of Lay Workers & Peer Education | Mobilizing members of the target population to serve as boundary spanners, credible sources of information, and role models. | Natural helpers in community with opinion leader status and availability to volunteer for training. | 5 |
| Using Imagery | Using artifacts that have a similar appearance to some subject. | Familiar physical or verbal images as analogies to a less familiar process. | 1 |

**Table S4:** Summary of calculated effectiveness scores and theoretical match between the identified behavioral change methods and determinants of the studies included in the review (n=15).

| **Author (year)** | **Effectiveness scores** | | **Theoretical match of determinants by identified Behavioral Change Methods (%)** | **Average  theoretical match** |
| --- | --- | --- | --- | --- |
|  | **Condom-use (%)** | **Psychosocial Determinants (%)** |  |  |
| Bull (2012) | 33.3% | 0% | Attitude / Outcome Expectations / Risk Perceptions  0.00%  Self-efficacy / Skills  0.00% | 0.00% |
| Clarke |  | 100% | Attitude / Outcome Expectations / Risk Perceptions  100%  Self-efficacy / Skills  50.0% | 75% |
| Hightow-Weidman (2018) | 33.3% |  | Knowledge is mentioned.  Underlying determinants unknown. |  |
| Ko (2013) | 33.3% |  | None |  |
| Swendeman (2024) | 33.3% |  | None |  |
| Neubaum (2014) |  | 66.7% | Attitude / Outcome Expectations / Risk Perceptions  100%  Self-efficacy / Skills  25.0% | 62.5% |
| Boonkaewwan (2020) |  | 50% | Attitude / Outcome Expectations / Risk Perceptions  50.0%  Self-efficacy / Skills  50.0% | 50.0% |
| Prybutok (2013) |  | 100% | Attitude / Outcome Expectations / Risk Perceptions  75.0%  Self-efficacy / Skills  25.0% | 50.0% |
| Jones (2012) | 100% |  | Attitude / Outcome Expectations / Risk Perceptions  0.00%  Self-efficacy / Skills  100% | 50.0% |
| Sun (2017) | 0% | 0% | Attitude / Outcome Expectations / Risk Perceptions  50.0%  Self-efficacy / Skills  50.0% | 50.0% |
| Hutchinson (2019) | 50% | 50% | Self-efficacy / Skills  28.57% | 28.57% |
| Whiteley (2018) | 100% | 50% | Self-efficacy / Skills  100% | 100% |
| Fernandez (2019) | 0% | 0% | Attitude / Outcome Expectations / Risk Perceptions  60.0% | 60.0% |
| Young (2013) |  | 100% | None |  |
| Bhandari (2024) |  | 100% | None |  |

Table S5: Summary of the quality assessment of the studies included in the review, based on the Quality Assessment Tool for Quantitative Studies by the Effective Public Health Practice Project (EPHPP).

| **First Author (Year)** | **Selection**  **Bias** | **Study**  **Design** | **Confounders** | **Blinding** | **Data Collection*** | **Withdrawals / Drop-out** | **Overall score** |
| --- | --- | --- | --- | --- | --- | --- | --- |
| Bull (2012) | Weak | Strong | Strong | Moderate | Moderate | Moderate | Moderate |
| Clarke (2020) | Moderate | Moderate | Weak | Moderate | Weak | Moderate | Weak |
| Hightow-Weidman (2018) | Weak | Strong | Strong | Moderate | Moderate | Moderate | Moderate |
| Ko (2013) | Weak | Moderate | Weak | Moderate | Moderate | Weak | Weak |
| Swendeman (2024) | Weak | Strong | Strong | Strong | Moderate | Moderate | Moderate |
| Neubaum (2014) | Weak | Strong | Strong | Moderate | Strong | Not applicable | Moderate |
| Boonkaewwan (2020) | Weak | Strong | Strong | Moderate | Strong | Weak | Weak |
| Prybutok (2013) | Weak | Strong | Strong | Moderate | Weak | Strong | Weak |
| Jones (2012) | Weak | Weak | Weak | Moderate | Strong | Weak | Weak |
| Sun (2017) | Weak | Strong | Strong | Moderate | Strong | Strong | Moderate |
| Hutchinson (2019) | Moderate | Moderate | Strong | Moderate | Strong | Weak | Moderate |
| Whiteley (2018) | Weak | Strong | Strong | Moderate | Strong | Strong | Moderate |
| Fernandez (2019) | Weak | Strong | Strong | Moderate | Weak | Weak | Weak |
| Young (2013) | Moderate | Strong | Weak | Moderate | Moderate | Weak | Weak |
| Bhandari (2024) | Weak | Strong | Strong | Moderate | Strong | Strong | Moderate |

*Note*. *According to EPHPP guidelines, studies are rated as “Weak” when the validity and reliability of measurement instruments are not reported. However, in this review, studies using behavioral outcomes (e.g. items measuring condom use at last sex or the number of sexual partners in last month) were rated as “Moderate”, as the reliability and validity of such measures can reasonably be assumed.

**Table S6:** Completed PRISMA 2020 checklist for the systematic review of social network based interventions promoting condom use among youth.

| Section and Topic | Item # | Checklist item | Location where item is reported |
| --- | --- | --- | --- |
| **TITLE** | | |  |
| Title | 1 | Identify the report as a systematic review. | Title page line 1 |
| **ABSTRACT** | | |  |
| Abstract | 2 | See the PRISMA 2020 for Abstracts checklist. | Abstract |
| **INTRODUCTION** | | |  |
| Rationale | 3 | Describe the rationale for the review in the context of existing knowledge. | Introduction, paragraph 3 onwards |
| Objectives | 4 | Provide an explicit statement of the objective(s) or question(s) the review addresses. | Introduction, final paragraph |
| **METHODS** | | |  |
| Eligibility criteria | 5 | Specify the inclusion and exclusion criteria for the review and how studies were grouped for the syntheses. | Methods, “Eligibility Criteria” |
| Information sources | 6 | Specify all databases, registers, websites, organisations, reference lists and other sources searched or consulted to identify studies. Specify the date when each source was last searched or consulted. | Methods, “Search Strategy” |
| Search strategy | 7 | Present the full search strategies for all databases, registers and websites, including any filters and limits used. | Methods, “Search Strategy” |
| Selection process | 8 | Specify the methods used to decide whether a study met the inclusion criteria of the review, including how many reviewers screened each record and each report retrieved, whether they worked independently, and if applicable, details of automation tools used in the process. | Methods, “Search Strategy” and “Eligibility Criteria” |
| Data collection process | 9 | Specify the methods used to collect data from reports, including how many reviewers collected data from each report, whether they worked independently, any processes for obtaining or confirming data from study investigators, and if applicable, details of automation tools used in the process. | Methods, “Search Strategy” and “Data Extraction” |
| Data items | 10a | List and define all outcomes for which data were sought. Specify whether all results that were compatible with each outcome domain in each study were sought (e.g. for all measures, time points, analyses), and if not, the methods used to decide which results to collect. | Methods, “Data Extraction” |
|  | 10b | List and define all other variables for which data were sought (e.g. participant and intervention characteristics, funding sources). Describe any assumptions made about any missing or unclear information. | Methods, “Data Extraction” |
| Study risk of bias assessment | 11 | Specify the methods used to assess risk of bias in the included studies, including details of the tool(s) used, how many reviewers assessed each study and whether they worked independently, and if applicable, details of automation tools used in the process. | Methods, “Quality Appraisal” |
| Effect measures | 12 | Specify for each outcome the effect measure(s) (e.g. risk ratio, mean difference) used in the synthesis or presentation of results. | Methods, “Data Extraction” and “Data Analysis” |
| Synthesis methods | 13a | Describe the processes used to decide which studies were eligible for each synthesis (e.g. tabulating the study intervention characteristics and comparing against the planned groups for each synthesis (item #5)). | Methods, “Data Analysis” |
|  | 13b | Describe any methods required to prepare the data for presentation or synthesis, such as handling of missing summary statistics, or data conversions. | Methods, “Data Analysis” |
|  | 13c | Describe any methods used to tabulate or visually display results of individual studies and syntheses. | Methods, “Data Analysis” |
|  | 13d | Describe any methods used to synthesize results and provide a rationale for the choice(s). If meta-analysis was performed, describe the model(s), method(s) to identify the presence and extent of statistical heterogeneity, and software package(s) used. | Methods, “Data Analysis” |
|  | 13e | Describe any methods used to explore possible causes of heterogeneity among study results (e.g. subgroup analysis, meta-regression). | Methods, “Data Analysis” |
|  | 13f | Describe any sensitivity analyses conducted to assess robustness of the synthesized results. | NA. |
| Reporting bias assessment | 14 | Describe any methods used to assess risk of bias due to missing results in a synthesis (arising from reporting biases). | NA. |
| Certainty assessment | 15 | Describe any methods used to assess certainty (or confidence) in the body of evidence for an outcome. | Methods, “Data Extraction”, and “Data Analysis” |
| **RESULTS** | | |  |
| Study selection | 16a | Describe the results of the search and selection process, from the number of records identified in the search to the number of studies included in the review, ideally using a flow diagram. | Results, “Study Selection” |
|  | 16b | Cite studies that might appear to meet the inclusion criteria, but which were excluded, and explain why they were excluded. | Results, “Study Selection” |
| Study characteristics | 17 | Cite each included study and present its characteristics. | Results, “Study Selection” |
| Risk of bias in studies | 18 | Present assessments of risk of bias for each included study. | Results, “Study Selection” and Table S5 |
| Results of individual studies | 19 | For all outcomes, present, for each study: (a) summary statistics for each group (where appropriate) and (b) an effect estimate and its precision (e.g. confidence/credible interval), ideally using structured tables or plots. | Table 2 |
| Results of syntheses | 20a | For each synthesis, briefly summarise the characteristics and risk of bias among contributing studies. | Table S5 |
|  | 20b | Present results of all statistical syntheses conducted. If meta-analysis was done, present for each the summary estimate and its precision (e.g. confidence/credible interval) and measures of statistical heterogeneity. If comparing groups, describe the direction of the effect. | Table 2 |
|  | 20c | Present results of all investigations of possible causes of heterogeneity among study results. | Table 2 & Results, “Study Selection” & Discussion, paragraph 8 |
|  | 20d | Present results of all sensitivity analyses conducted to assess the robustness of the synthesized results. | NA |
| Reporting biases | 21 | Present assessments of risk of bias due to missing results (arising from reporting biases) for each synthesis assessed. | Table S5 |
| Certainty of evidence | 22 | Present assessments of certainty (or confidence) in the body of evidence for each outcome assessed. | Table 2 |
| **DISCUSSION** | | |  |
| Discussion | 23a | Provide a general interpretation of the results in the context of other evidence. | Discussion, paragraph 2 onwards |
|  | 23b | Discuss any limitations of the evidence included in the review. | Discussion, paragraph 8 |
|  | 23c | Discuss any limitations of the review processes used. | Discussion, paragraph 8 |
|  | 23d | Discuss implications of the results for practice, policy, and future research. | Discussion, paragraph 2 onwards |
| **OTHER INFORMATION** | | |  |
| Registration and protocol | 24a | Provide registration information for the review, including register name and registration number, or state that the review was not registered. | Methods, paragraph 1 |
|  | 24b | Indicate where the review protocol can be accessed, or state that a protocol was not prepared. | Methods, paragraph 1 |
|  | 24c | Describe and explain any amendments to information provided at registration or in the protocol. | Data extraction, paragraph 2 |
| Support | 25 | Describe sources of financial or non-financial support for the review, and the role of the funders or sponsors in the review. | Acknowledgements |
| Competing interests | 26 | Declare any competing interests of review authors. | Conflicts of Interest |
| Availability of data, code and other materials | 27 | Report which of the following are publicly available and where they can be found: template data collection forms; data extracted from included studies; data used for all analyses; analytic code; any other materials used in the review. | NA |
